# Power of neutrality tests for detecting natural selection

**DOI:** 10.1093/g3journal/jkad161

**Published:** 2023-07-22

**Authors:** Tomotaka Tanaka, Toshiyuki Hayakawa, Kosuke M Teshima

**Affiliations:** Graduate School of System Life Science, Kyushu University, Fukuoka 819-0395, Japan; Graduate School of System Life Science, Kyushu University, Fukuoka 819-0395, Japan; Faculty of Arts and Science, Kyushu University, Fukuoka 819-0395, Japan; Department of Biology, Faculty of Science, Kyushu University, Fukuoka 819-0395, Japan

**Keywords:** natural selection, power, simulation

## Abstract

Detection of natural selection is one of the main interests in population genetics. Thus, many tests have been developed for detecting natural selection using genomic data. Although it is recognized that the utility of tests depends on several evolutionary factors, such as the timing of selection, strength of selection, frequency of selected alleles, demographic events, and initial frequency of selected allele when selection started acting (softness of selection), the relationships between such evolutionary factors and the power of tests are not yet entirely clear. In this study, we investigated the power of 4 tests: Tajiama's *D*, Fay and Wu's *H*, relative extended haplotype homozygosity (rEHH), and integrated haplotype score (iHS), under ranges of evolutionary parameters and demographic models to quantitatively expand the understanding of approaches for detecting selection. The results show that each test detects selection within a limited parameter range, and there are still wide ranges of parameters for which none of these tests work effectively. In addition, the parameter space in which each test shows the highest power overlaps the empirical results of previous research. These results indicate that our present perspective of adaptation is limited to only a part of actual adaptation.

## Teaser

Tanaka et al. investigated the power of four tests of neutrality: Tajiama’s D, Fay and Wu’s H, rEHH, and iHS, under ranges of evolutionary parameters and demographic models. The results show that each test detects selection within a limited parameter range, and there are still wide ranges of parameters for which none of these tests work effectively.

## Introduction

Living organisms have evolved under the effects of natural selection. Therefore, identifying the targets of natural selection is essential to understanding when, how, and why organisms evolve into what they are today. With the development of sequencing technologies, massive amounts of genomic data have become available (Goodwin *et al.* 2016; Shendure *et al.* 2017). Consequently, many methods for detecting natural selection using genomic data have been developed (Vitti *et al.* 2013; Fan *et al.* 2016; Rees *et al.* 2020). Tests for detecting natural selection from population genetic data can be classified into 2 groups. The first group focuses on site frequency spectrum (SFS), and the second utilizes the homozygous haplotype structure. Examples of widely used tests in SFS-based methods include Tajima's *D* (Tajima 1989) and Fay and Wu's *H* (Fay and Wu 2000). The effect of selection causes a reduction in the genetic diversity around the target site and therefore distorts the frequency spectrum. Excess low-frequency variants than expected under neutrality can be considered a signature of beneficial mutations. Tajima's *D* has detected this signature. Selection can also cause an excess of high-frequency variants because linked neutral variants hitchhike on the target site of selection and reach a high frequency. Fay and Wu's *H* detects this departure from neutral expectations. In order to capture an excess of high-frequency derived allele, unfolded SFS is required in calculation of Fay and Wu's *H* statistic. Haplotype-based tests include relative extended haplotype homozygosity (rEHH) (Sabeti *et al.* 2002) and integrated haplotype score (iHS) (Voight *et al.* 2006). Because the number of selected alleles rapidly increases in a population, there is less time for recombination to break down linkage disequilibrium (LD) around the target of selection. rEHH and iHS detect excess homozygous tracts around the target site as a signature of positive selection. Since these methods detect natural selection by capturing different signatures of genomic diversity (e.g. a skew in SFS and long-range LD), different statistics might be useful in different parts of the parameter space. Indeed, different methods have uncovered different candidate genes (Sabeti *et al.* 2006; Nielsen *et al.* 2007).

Only a part of the natural selection that has actually occurred is currently observed. Sabeti *et al.* (2006) have shown that different methods detect selection in different evolutionary time windows. In addition, Zhai *et al.* (2009) pointed out that methods based on population genetic data detect signatures of selection in a relatively narrow time window compared with tests based on comparative genetic data because they mainly detect ongoing selection. Although some previous studies have discussed the relationship between power and timing of selection (Simonsen *et al.* 1995; Sabeti *et al.* 2006), they have not been quantitatively examined. Thus, the time range of natural selection that can be detected remains unclear.

The power of tests for detecting selection depends not only on the timing of selection but also on other factors, such as the strength of selection and frequency of selected alleles. Although some previous studies (Voight *et al.* 2006; Zeng *et al.* 2006; Huff *et al.* 2010; Ma *et al.* 2015; Vatsiou *et al.* 2016) have examined the theoretical background of detectable conditions, the parameter sets examined were limited, and different studies used different parameter sets. Demographic events, such as population expansion and bottlenecks, have also considerable effects on detecting selection (Fu 1997; Ramos-Onsins and Rozas 2002; Depaulis *et al.* 2003; Nielsen 2005). Several previous studies have investigated the robustness of these 4 tests, Tajima's *D*, Fay and Wu's *H*, rEHH, and iHS, against demographic events (Simonsen *et al.* 1995; Depaulis *et al.* 2003; Ramírez-Soriano *et al.* 2008; Huff *et al.* 2010; Li 2011; Li *et al.* 2012; Ferrer-Admetlla *et al.* 2014; Vatsiou *et al.* 2016). For example, Ferrer-Admetlla *et al.* (2014) examined the distribution of these 4 test statistics under various population expansion and bottleneck models. They found that both haplotype-based statistics and SFS-based statistics are not generally robust, and the SFS-based statistics are more affected by population expansion and bottlenecks than haplotype-based statistics. However, power and false-positive rates (FPRs) have not been examined in a wide range of parameter spaces; thus, it is still unclear whether we can distinguish between selection and demography. Demographic events also affect the evolutionary period when selection can be detected. For example, it has been pointed out that selection before a bottleneck and selection after a bottleneck leave different SFS patterns (Li *et al.* 2012). Therefore, the effects of demographic events should be examined.

The initial frequency of the target allele when selection started acting, or the softness of selection, is another factor. When selection acts on standing variations (SSV), the selected region shows a different diversity pattern compared with a region where selection for de novo mutations (SDN) occurs (Innan and Kim 2004; Przeworski *et al.* 2005). As previous research (Huff *et al.* 2010; Ferrer-Admetlla *et al.* 2014; Garud *et al.* 2015) shows, tests generally have less power in detecting SSV than in detecting SDN. However, it is not clear how the initial frequency of selected allele changes the time range that these tests can detect selection. Because SSV is widespread in many species, such as humans and *Drosophila* (Garud *et al.* 2015; Schrider and Kern 2017), the power of detecting soft selection should also be carefully considered.

Despite these several studies, the conditions of selective sweeps that have been observed are not yet entirely clear. That is why it is not easy to comprehensively understand the nature of selective sweeps. Given the fact that there is an increase in research attempting to detect selective sweeps for many species including nonmodel species (reviewed in Ekblom and Galindo 2011; Savolainen *et al.* 2013; Ellegren 2014; Xiang-Yu *et al.* 2016; Weigand and Leese 2018), it is worthwhile to examine the detectability under a wide range of parameter space in which the tests work effectively. In this study, the power of tests for detecting selective sweeps was comprehensively investigated by conducting simulations using a range of evolutionary parameters and demographic models. As seen above, the power of tests for detecting selective sweeps should be considered as a function of evolutionary factors, such as the strength of selection, the timing when selection acted, the demography of a population, and the softness of selection. By obtaining the power for detecting selective sweeps considering various factors, here we quantitatively expand our understanding of approaches for detecting selective sweeps. First, the power of the tests was evaluated using relatively simple models. By investigating the range and combination of parameters where selective sweeps can be detected, we confirmed which portions of evolutionary changes were observed. Subsequently, the power for detecting selective sweeps during the course of human evolution was assessed. It was shown that the parameter space in which current tests can detect selective sweeps is quite limited and that our present perspective of selective sweeps is limited to only a part of actual selective sweeps.

## Methods

### Demographic models

In this study, the power and error rate of neutrality tests were investigated using 3 basic demographic models and estimated human demographic models. In all models, diploid populations were assumed. The first model was a constant population model with *N*_0_ = 5,000, where *N*_0_ is the current population size. Throughout this study, the generation time was set to 20 years. The next model was the expansion model. After the expansion, the size of the current population becomes 10 times larger than that of the ancestral population. Namely, $N_{0}/N_{1}=10$, where *N*_1_ is the ancestral population size prior to the expansion. We varied the population size and timing of demographic events so that all models had the same effective population size as the constant model (*N_e_* = 5,000). Specifically, 3 cases were considered: (1) *N*_1_ = 4,770 and *t*_1_ = 0.1, (2) *N*_1_ = 4,330 and *t*_1_ = 0.3, and (3) *N*_1_ = 3,890 and *t*_1_ = 0.5, where *t*_1_ is the time when expansion occurred (in unit of *N* generation). In the bottleneck model, the current population size was 20 times larger than the ancestral population size during a bottleneck. Namely, we set $N_{0}/N_{b}=20$ and the duration of the bottleneck was 500 generations, where *N_b_* is the population size during the bottleneck. Three combinations of the bottleneck population size and the timing of the bottleneck, with the same effective population size as previous models (*N_e_* = 5,000), were considered: (1) *N_b_* = 427 and *t_b_* = 0.05, (2) *N_b_* = 418 and *t_b_* = 0.2, and (3) *N_b_* = 401 and *t_b_* = 0.5, where *t_b_* is the time at which the bottleneck ended (in *N* generation). In addition to the aforementioned basic demographic models, models that are plausible for human evolution were also considered. The models estimated by Schaffner *et al.* (2005) (Supplementary Fig. 1) and a simplified version of the model estimated by Li and Durbin (2011) (Supplementary Fig. 2) were used.

### Coalescent simulation

The program *mbs* (Teshima and Innan 2009) was used to generate the sample sequences. To investigate the power and error rate of the tests for selection acting on variants at a certain allele frequency, frequency trajectories conditioned on the current allele frequency were simulated. A backward-in-time simulation was used under the constant population model, and rejection sampling was applied under the models in which the population size changed. When the power and error rate of tests for the selection of variants that arose at a certain age were assessed, a forward-in-time simulation was used to generate the allele frequency trajectory. The allele frequency trajectories were generated by performing simulations based on the Wright–Fisher model. The scripts are available on our GitHub site (https://github.com/ttomo3535/power_of_neutrality_tests). The sample sequences were then generated using *mbs* based on the simulated frequency trajectories.

**Fig. 1.**
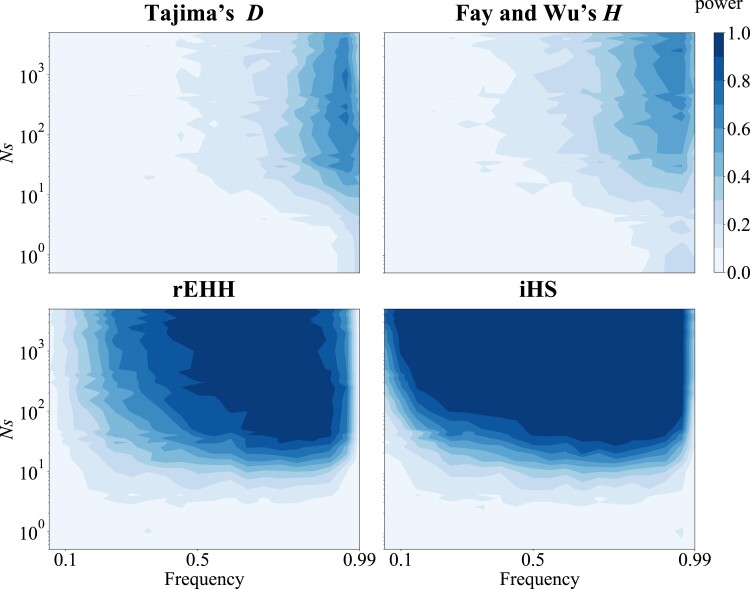
The power of 4 tests against the strength of selection and allele frequency. The power of Tajima's *D*, Fay and Wu's *H*, rEHH, and iHS under the constant population model is plotted against the strength of selection (*Ns*, *y*-axis) and allele frequency in the current population. Selection acted on de novo mutation.

**Fig. 2.**
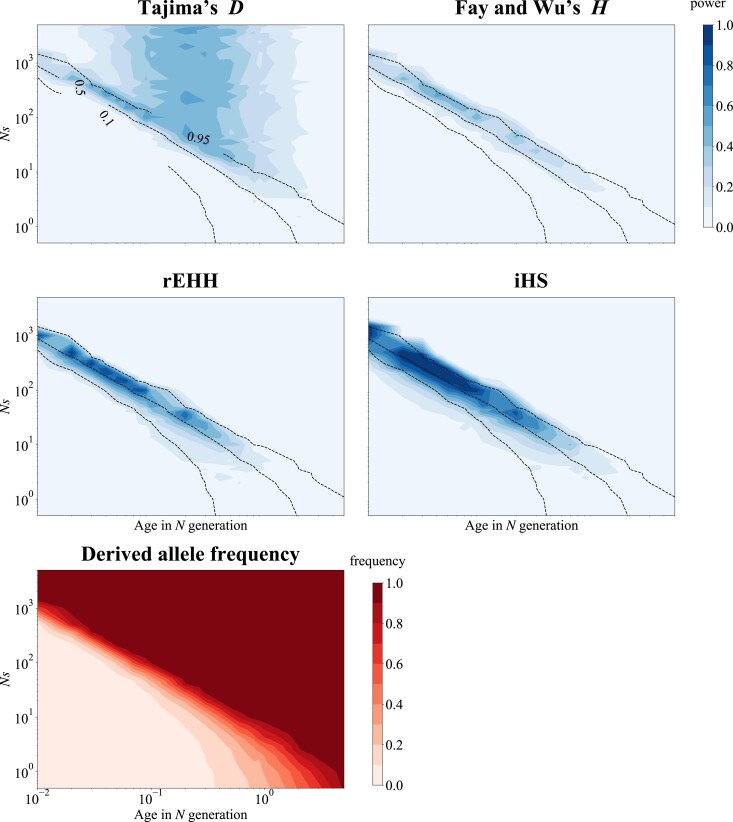
The power of 4 tests and the current allele frequency against the strength of selection and allele age. The power of Tajima's *D*, Fay and Wu's *H*, rEHH, and iHS under the constant model is plotted against the strength of selection and age of the derived allele (top and middle). The dotted contour plot represents allele frequency in the current population. The average of the current allele frequency is plotted against the strength of selection and age of the derived allele (bottom). Selection acted on de novo mutation.

**Fig. 3.**
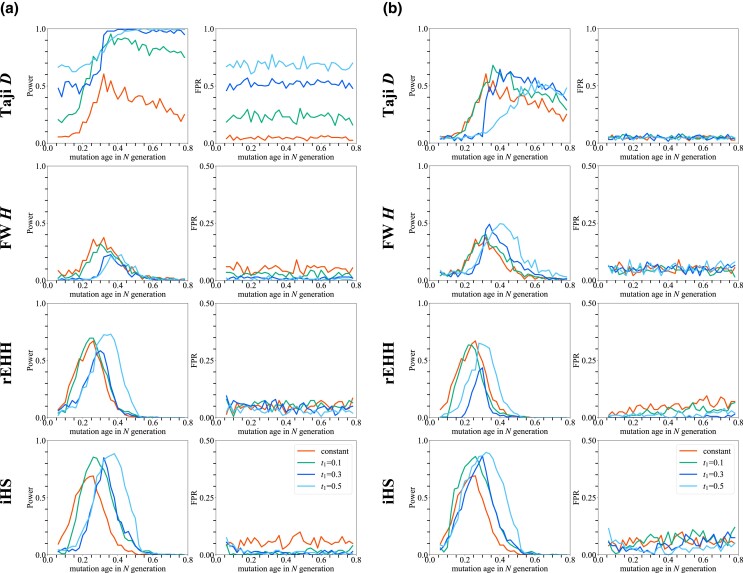
The power and FPR of 4 tests under expansion models. The power (left columns) and FPR (right columns) of 4 tests are plotted against the age of mutation. The constant population model and 3 expansion models with *t*_1_ = 0.1, 0.3, and 0.5, respectively, where *t*_1_ is the timing of expansion in a unit of *N* generations were used. The selection coefficient *s* is set to be 0.005. a) Null distribution was generated under the standard neutral model with *N* = 5,000. b) Null distribution was generated under the true demographic mode.

**Fig. 4.**
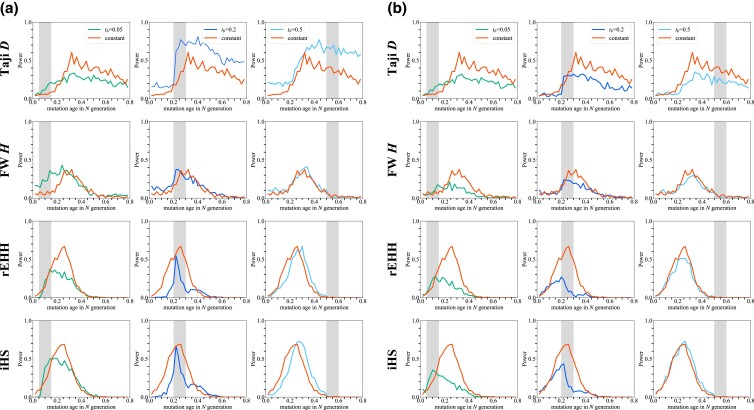
The power of 4 tests under bottleneck models. The power of 4 tests is plotted against the age of mutation. The constant population model and 3 bottleneck models with *t_b_* = 0.05, 0.2, and 0.5, respectively, where *t_b_* is the time when the bottleneck ended in a unit of *N* generations were used. The selection coefficient *s* is set to be 0.005. a) The power and FPR were calculated with null distribution generated under the standard neutral model (*N* = 5,000). b) Null distribution generated under the true demographic model was used. The duration of the bottleneck is illustrated by the shaded area.

**Fig. 5.**
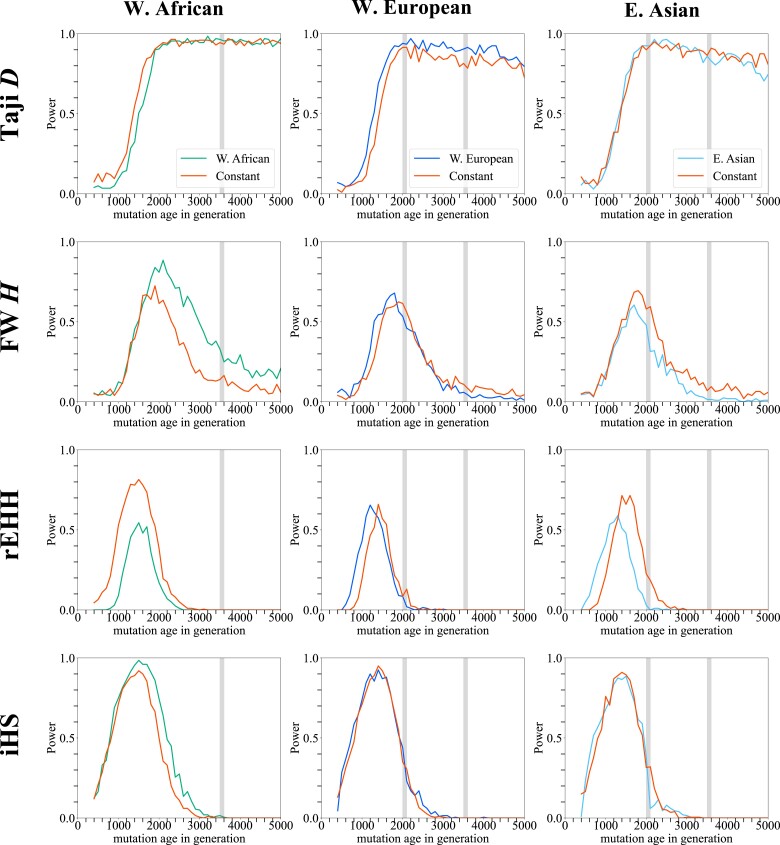
The power of 4 tests under human demographic models. The power of 4 tests is plotted against the age of mutation. Models plausible for W. African, W. European, and E. Asian populations (left to right) and under the constant population model with *N* = 16,000, 13,000, and 12,000 (left to right) were used. The duration of the bottleneck is highlighted in gray. The selection coefficient *s* is set to be 0.005. Selection acted on de novo mutation. The null distributions were generated under each demographic model.

**Fig. 6.**
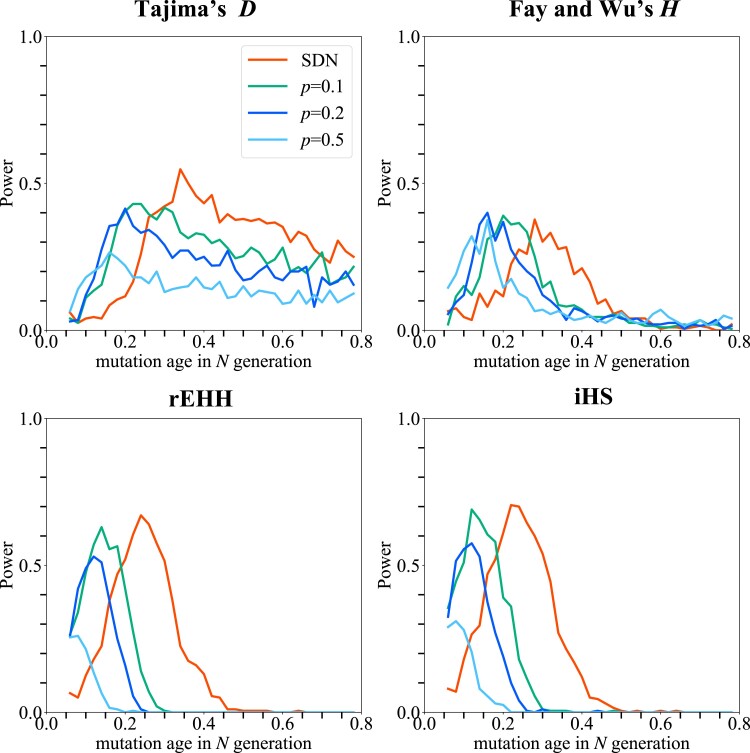
The power of 4 tests for detecting SSV. The power of 4 tests is plotted. Selection works on de novo mutation (SDN) and SSV (*P* = 0.01, 0.1, 0.2, and 0.5). The selection coefficient *s* is set to be 0.005.

To simulate the allele frequency trajectories affected by standing variations, both backward and forward simulations were used. Frequency trajectories were conditioned on allele frequencies when selection started. Backward simulation was used to generate ancestral neutral trajectories, and forward simulation was used to generate trajectories after the start of selection. The effect of SSV was investigated by changing the initial frequency of the selected allele *p*_1_ when selection started. The length of the simulated region was 10 kb when the SFS-based statistics were analyzed. The target site of selection was located at the center of the 10-kb region. When haplotype-based statistics were investigated, 500-kb regions were simulated. The target site for selection was located at the edge of the simulated region. *μ* = 1.0 × 10^−8^ and *r* = 1.0 × 10^−8^, where *μ* is the mutation rate per site per generation and *r* is the recombination rate per site per generation. One hundred twenty chromosomes were sampled from a population. First a wide range of selection coefficients of *s* was used (0.0001–1) (Figs. 1 and 2). Then, *s* = 0.005 was used as the representative value of weak selection, assumed relatively weak selection (*Ns* = 25) to better understand the effect of demographic events and softness of selection (Figs. 3–6; Supplementary Figs. 6–11).

### Test statistics

The power and error rate of 2 SFS-based tests, Tajima's *D* (Tajima 1989) and Fay and Wu's *H* (Fay and Wu 2000), and 2 haplotype-based tests, rEHH (Sabeti *et al.* 2002) and iHS (Voight *et al.* 2006), were investigated. Tajima's *D* and Fay and Wu's *H* were calculated using our own script, and the extended haplotype homozygosity (EHH) and iHS statistics were calculated using the *rehh* package (Gautier *et al.* 2017). EHH statistics were calculated at 0.025 cM (25,000 bp) from the target site in the constant population model with *N*_0_ = 5,000. In the expansion models, because the degree of LD decay differs depending on the degree of expansion, the distance for rEHH calculation was set up so that roughly the same number of SNP sites was included within the region. EHH was calculated at the site 25,000 bp from the target for the model with (*N*_1_, *t*_1_) = (4,770, 0.1), at 20,000 bp from the target for (*N*_1_, *t*_1_) = (4,330, 0.3), and at 15,000 bp from the target for (*N*_1_, *t*_1_) = (3,890, 0.5); thus, the number of segregating sites was approximately the same as that under the constant model. In the human model, EHH was calculated at 10,000 bp from the target site for the W. African model, at 20,000 bp from the target for the European and E. Asian models (Schaffner *et al.* 2005), and at 20,000 bp from the target for European and Asian models (Li and Durbin 2011).

Null distributions of SFS-based tests were generated under demographic models using the program *ms* (Hudson 2002). The program *mbs* was used to generate the null distribution of haplotype-based tests. Power is defined as the proportion of simulations that fall at the 5% end of the null distribution (1-sided). FPR is defined as the proportion of simulated data sets generated under a neutral model that rejects the neutral null hypothesis at the 5% significance level. The power and FPR were evaluated based on 200 replicates.

## Results

### Power to detect selection under constant population model

First, the power to detect selection acting on de novo mutations under the constant population model was investigated. The powers of the 4 tests, conditional on the current-derived allele frequencies and the strength of selection, are shown in Fig. 1. The power of SFS-based tests (Tajima's *D* and Fay and Wu's *H*) depends on the current derived allele frequencies and takes the highest power when the allele frequency is 90–95%. However, the power decreases when the derived allele frequency is almost 100% because genetic diversity is removed by the effect of selective sweep (Zeng *et al.* 2006). Across a wide range of allele frequencies, the power of haplotype-based tests (rEHH and iHS) was higher than that of SFS-based tests. However, when the derived allele frequency was near 100%, the power of haplotype-based tests was lower than that of SFS-based tests (Supplementary Fig. 3). This reduction in the power of haplotype-based tests results from their inability to calculate statistics. Often, the ancestral haplotype was not involved in a sample when the derived allele frequency was very high, and statistical values could not be calculated in such a case. Regardless of the test, it was difficult to detect selection when *Ns* < 10. From these results, it can be concluded that positive selection can be detected if the selection coefficient is reasonably strong by appropriately combining the statistical tests.

The power conditional on the age of the derived allele is shown in Fig. 2. The results for Fay and Wu's *H*, rEHH, and iHS showed similar patterns. rEHH and iHS can detect positive selection only when the selected allele is segregated (Sabeti *et al.* 2002; Voight *et al.* 2006). Fay and Wu's *H* detects selection when the selected allele is close to fixation or during a short period after fixation (Zeng *et al.* 2006). Therefore, the parameter range to detect selection for these tests is limited to combinations in which the derived allele frequencies are moderate to high (Fig. 2). Tajima's *D*, on the other hand, can detect selection even after fixation. It seems that the power depends on the age of the derived allele and peaks around 0.2*N* generations ago. Thus, it can be argued that Tajima's *D* can detect selection under a wider range of parameters, although haplotype-based tests have higher power. These results indicate that the candidates for natural selection that have been detected are only a part of the selection. There are still wide ranges of parameters for which none of these tests work effectively, such as recent weak selections.

### Effect of demographic changes

The effects of demographic events on the power and error rates of the tests were investigated. Two types of null distributions were considered to calculate power and error rates. The first was generated using a standard neutral model with *N* = 5,000. The second one was generated under the same demographic model as the one under which selection was simulated (true demographic model) to assess if we could mitigate the effect of demographic events by estimating the demographic history.

Owing to population expansion, the power and false positives of Tajima's *D* greatly increased (Fig. 3a). Fay and Wu's *H* showed a decrease in power (Fig. 3; Supplementary Fig. 4a). The degree of reduction in power depends on the timing of expansion (Supplementary Fig. 5). Haplotype-based tests showed an increase in FPR especially when the frequency of neutral allele is high (Supplementary Fig. 4a). Although the power of rEHH was less affected than other tests, a slight increase in FPR was observed. iHS showed an increase in power but the FPR also increased. Haplotype-based tests show the same or lower level of FPR against age of the mutation than those under the constant population model (Fig. 3). This is because the current frequency of neutral allele that arose during the period between 0 and 0.8 *N* generations ago is not high enough for haplotype-based tests to show an increase in FPR (Supplementary Fig. 6b). However, these effects of expansion can be mitigated if a null distribution is generated by considering demography (Fig. 3b; Supplementary Fig. 4b). It was shown that the FPR of Tajima's *D* decreases if a null distribution is generated under true demography, although the power decreases at the same time (Supplementary Fig. 4b). On the contrary, the power of Fay and Wu's *H* rather increased (Fig. 3b; Supplementary Fig. 4b). The FPR of rEHH was suppressed while retaining its power. iHS increased in power, which is consistent with a previous study (Huff *et al.* 2010). Thus, except for Tajima's *D*, we can mitigate the effect of expansion by considering the demography. Additionally, for Fay and Wu's *H* and haplotype-based tests, the peak of power shifted toward older age. The reason for this is that it takes more time for a selected allele to reach a moderate to high frequency, which is these 3 tests can work reasonably (Supplementary Fig. 6a).

When a population experienced a recent bottleneck, the power of haplotype-based tests to detect advantageous mutations that arose before or during the bottleneck phase decreased (Fig. 4a; Supplementary Fig. 7a). The reduction in the power of rEHH and iHS was caused by the fixation of the selected alleles. Because rEHH and iHS cannot be implemented after fixation, the chances of detecting selection that started acting during or before the bottleneck decreased. Contrary to the cases of population expansion, the effects of bottlenecks cannot be mitigated even if the demography is taken into consideration when calculating the null distribution (Fig. 4b; Supplementary Fig. 7b). Tajima's *D* also showed a decrease in power when a population experienced a recent bottleneck (Fig. 4a and b). Although the power of Fay and Wu's *H* increased, FPR also increased (Supplementary Figs. 8 and 9). When the null distribution generated under true demography was used to mitigate the increase in FPR, the power to detect selection that started before or during the bottleneck phase decreased (Fig. 4b). Therefore, if a bottleneck occurs after or during the detectable time range of these tests, selection before and during the bottleneck phase is difficult to detect, regardless of the demographic model of the null distribution.

Power was assessed by incorporating human demographic models estimated by Schaffner *et al.* (2005) to understand the joint effect of demographic events. A null distribution was generated for each demographic model. The power under the estimated demographic models was compared with that under a constant population with the same effective size to assess the effects of demography when several events were combined (Fig. 5). In all 3 populations, Tajima's *D* shows comparable power with that under the constant population. On the contrary, in the African population, Fay and Wu's *H* shows higher power than that under the constant population. This increase in power is due to the expansions that the African population experienced (Fig. 3). For the European and Asian populations, because they experienced population constriction, the effects of expansion are mitigated and the power of Fay and Wu's *H* becomes comparable with that under the constant populations. The Asian population model shows a reduction in power owing to the more severe bottleneck that they experienced. iHS shows a level of power comparable with that under the constant population. This is because the timing of the bottleneck is older than the detectable time range.

### Power for detecting SSV

Next, the power to detect soft sweeps was investigated. The power of the tests for a derived allele with frequency *p* at *t* generations ago was assessed (Fig. 6). Compared with the power for de novo mutation (SDN), 2 distinctive patterns were observed in the case of SSV. First, the peak of power shifted toward younger age. When the initial frequency *p* is high, less time is required to reach a moderate to high frequency, which is these 4 tests can work reasonably. Therefore, when the initial frequency of standing variation is high, the detected alleles are limited to young alleles compared with those when *p* is small. Second, a reduction in power was observed. As the initial frequency *p* increases, the more diverse haplotypes selected alleles are associated with (Przeworski *et al.* 2005; Hermisson and Pennings 2017). This mitigates the reduction in diversity caused by selective sweep. Thus, the power is maintained only within a short period if *p* is high. The power of Tajima's *D*, rEHH, and iHS decreased as *p* increased, as suggested in a previous study (Ferrer-Admetlla *et al.* 2014). The detectable time span also narrowed. In contrast, the power of Fay and Wu's *H* did not change significantly regardless of the initial frequency, although the FPR increased (Supplementary Fig. 11).

We also demonstrated how power changes with the joint effect of bottlenecks and SSV (Table 1). It was assumed that selection started 2,000 generations ago when the Europe–Asia split bottleneck began under the E. Asian population model. To understand the effect, 4 cases were compared: SDN in the constant population model, SSV in the constant population, SDN in the E. Asian population model, and SSV in the E. Asian population model. For SSV, selection started when the derived allele frequency was 0.1. The results are shown in Table 1. In the case of (constant + SDN), SFS-based tests show higher power than those of haplotype-based tests, because the Europe–Asia split occurred at a bit older age than the time range that haplotype-based tests show its highest power. When selection acts on standing variations (constant + SSV), all 4 tests show a reduction in power. When a population experiences a bottleneck, the power for Fay and Wu's *H*, rEHH, and iHS becomes lower than those under a constant population. When selection acts on standing variations (bottleneck + SSV), the power for all tests decreases further. Therefore, under the demographic history plausible for human evolution, the most recent bottleneck imposed a basic limit on the detection of selection except for Tajima's *D*. If the selection acts on standing variations, the limitation becomes even more severe for all 4 tests.

**Table 1.** The power of 4 tests.

	Constant + SDN	Constant + SSV	E. Asian + SDN	E. Asian + SSV
Tajima's *D*	0.935	0.495	0.95	0.4
Fay and Wu's *H*	0.33	0.115	0.265	0.095
rEHH	0.13	0	0.005	0
iHS	0.205	0	0.08	0.025

The power of 4 tests (1) when selection works on de novo mutation under the constant population model (*N* = 10,000), (2) when selection works on standing variation under the constant model, (3) when selection works on de novo mutation under the model for E. Asian population, and (4) when selection started acting on standing variation (*P* = 0.1) under the model for E. Asian population (left to right column). Selection started 2,100 generations ago when the Europe-Asia split bottleneck occurred.

### Comparison with the empirical data

**Fig. 7.**
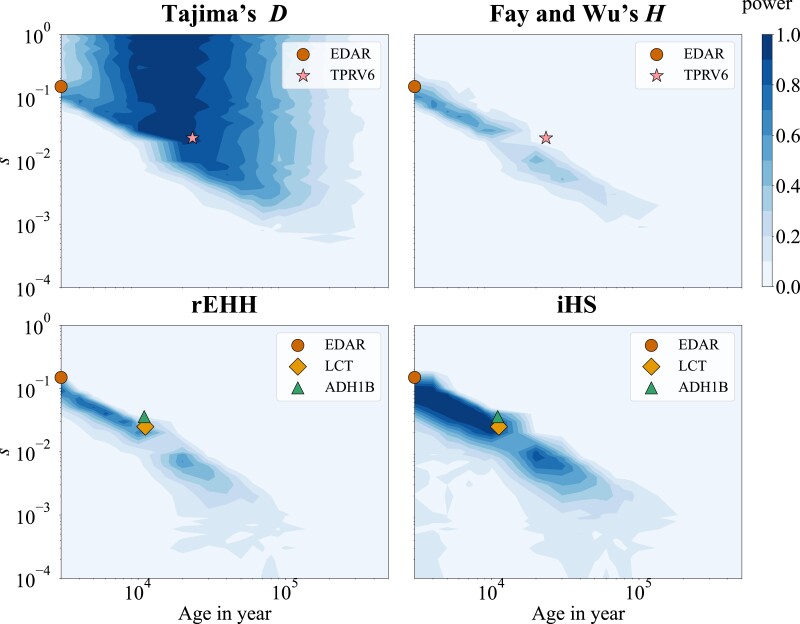
The power of 4 tests and the empirical data. The power of 4 tests is plotted. The simplified model for Europe and Asian population model (Li and Durbin 2011) is used. Selection acts on de novo mutation. The ages and selection coefficients for 4 candidate genes estimated by Peter *et al.* (2012) are also plotted.

Finally, to confirm our simulation results, we compared the results of the empirical analysis with those of our simulation. Hawks *et al.* (2007) estimated the ages of candidate genes for natural selection and claimed that human adaptation has accelerated recently. We assumed a constant population model with N=10,000 and set s=0.022 for the African population and s=0.034 for the European population, in accordance with Hawks *et al.* (2007). The power of the rEHH was then estimated. The power distribution of the rEHH calculated in this study and the ages of candidate genes estimated by Hawks *et al.* (2007) are plotted in Supplementary Fig. 12. As shown in the figure, the estimated ages overlapped with the power distribution. It is possible that the skewed distribution of estimated ages was caused by the power distribution of the tests. Peter *et al.* (2012) estimated evolutionary parameters of several candidate genes. We selected 4 candidate genes for which the selection acted as they arose. The 4 candidate genes were chosen based on 2 criteria: selection acted on de novo mutation and the selected allele had not yet reached fixation. They were plotted on a contour plot of the power of the tests (Fig. 7). In this contour plot, power was calculated using European and Asian human demographic models (Li and Durbin 2011). As shown in the figure, the age and strength of selection for these candidate genes overlapped in the area where each test had the highest power. Altogether, these results indicate that the currently observed adaptations by those traditional tests are restricted to the parameter spaces in which the signatures of selection can be detected. To further expand our understanding of adaptations, developing new test statistics to target the parameter space of selection that those tests miss or combining multiple tests are expected.

## Discussion

In this study, we investigated the power of tests of neutrality that utilized within-population genetic data. Most of the previous studies that focused on the efficiency of tests analyzed the power conditioned on allele frequency in order to extract candidate sites of selection from genomic data (Voight *et al.* 2006; Zeng *et al.* 2006; Huff *et al.* 2010; Ma *et al.* 2015; Vatsiou *et al.* 2016). To understand the relative contribution of selection to the mechanisms of evolution, we also focused on the power conditioned on the timing when selection started. Through this study, it was shown that each neutrality test has different feasible conditions: the timing and strength of selection. It was also shown that each test detected selections within a limited parameter range. This means that we can observe only a limited part of the sweeps actually occurred. For example, Tajima's *D* is useful for detecting adaptations that occur within a certain time range and reach some high frequency. Fay and Wu's *H*, rEHH, and iHS are only useful for recent adaptation for a limited range of *Ns*. Sabeti *et al.* (2006) roughly estimated the time range that these tests can detect selections: Tajima's *D* detects selection within 250,000 years; Fay and Wu's *H* detects selection within 80,000 years; and rEHH and iHS detect selections within 30,000 years, while admitting that many instances of selection are not likely to be detected by any currently proposed methods. Our results basically agree with these estimations; these tests detect selection within roughly 100,000 years (1 in a unit of *N* generations). Furthermore, this study clarified the combinations of parameters for which detections are possible. Thus, it was shown that selections detectable by each method are confined to a limited number of candidates that occurred in a certain parameter combination.

As previously suggested (Sabeti *et al.* 2006), some sweeps cannot be detected with current tests that utilize within-population polymorphism data. For example, it is still difficult to detect recent weak selections. Although rEHH and iHS detect recent sweeps relatively efficiently, they cannot detect adaptations once the selected alleles are fixed in a population. Tajima's *D* can detect selection even after fixation; however, its detectable time range is still limited. Thus, it should be noted that many true sweeps were absent from the previous empirical studies that used those tests to detect adaptions. Selection of segregating SNP at moderate frequencies can reasonably be detected (Fig. 1; Voight *et al.* 2006; Ferrer-Admetlla *et al.* 2014); however, it should be noted that these detections are nothing but a snapshot of adaptive alleles occurring during the course of evolution. It was demonstrated that selection that had started before the bottleneck was difficult to detect. Crisci *et al.* (2013) and Pavlidis and Alachiotis (2017) evaluated type 1 and 2 error rates of SFS-based methods (SweepFinder, SweeD, and SweepFinder2) and LD-based methods (OmegaPlus, iHS) under ranges of bottleneck models. However, how the timing of a bottleneck affects the evolutionary period when selection can be detected was not evaluated. We showed that not only type 2 error increases due to a recent bottleneck but also more specifically selection after a recent bottleneck is hard to detect. These results are consistent with a previous study (Schlamp *et al.* 2016). They showed that iHS is less accurate than other haplotype-based tests (H12 and H) and SFS-based tests (Composite likelihood ratio test and Tajima's *D*) in identifying selective sweeps by analyzing domesticated dogs, since they experienced recent bottlenecks during the process of domestication. We demonstrated that this is actually the case because selected alleles are easily fixed and iHS cannot detect those completed sweeps when a population experienced a recent bottleneck.

In empirical studies, several points should be noted. As we showed, some effects of demography on power can be mitigated by using null distribution generated under the correct demography. However, since the true demography is usually unknown, an inferred demographic history must be used. It should be noted that estimation error in inferring demographic history would result in an increase in false positives or a decrease in power. In addition, the target site is usually unknown in empirical studies. If tests are conducted for a close but different site, they will show even less power than if they were conducted on the true target site.

Because the neutrality tests investigated in this study do not have uniform sensitivity over the entire parameter range, it must be noted that the detection results do not necessarily reflect the true history of adaptation. One way to overcome this issue is to incorporate test statistics utilizing divergence between populations. Since those tests will detect selection even after the fixation of advantageous alleles (Lewontin and Krakauer 1973; Weir and Cockerham 1984; Akey *et al.* 2002; Kimura *et al.* 2007; Sabeti *et al.* 2007; Tang *et al.* 2007; Chen *et al.* 2010), a combination of tests utilizing within-population statistics and between population statistics may work complementarily (Grossman *et al.* 2010; Grossman *et al.* 2013; Utsunomiya *et al.* 2013; Ma *et al.* 2015; González-Rodríguez *et al.* 2016). Another possible way to overcome this issue is to integrate test statistics into inference methods, such as approximate Bayesian computation and machine learning (ML) framework, because different statistics perform differently depending on the parameters. In the previous studies, ML methods utilizing within-population statistics as feature values have been developed (Ronen *et al.* 2013; Schrider and Kern 2016; Schrider and Kern 2018) and indeed showed higher power than that of test based on a single statistic (Ronen *et al.* 2013). However, the aim of ML methods is to predict and classify genomic regions accurately rather than to understand the role of evolutionary processes. On the contrary, investigating the power of each test statistic can uniquely contribute to the understating of the relationship between the pattern of genomic diversity and evolutionary processes.

In this study, we did not investigate the effects of window sizes on power. As previous study showed, window size certainly changes the performance of tests (Ferrer-Admetlla *et al.* 2014). In fact, some machine learning methods vary window sizes to maximize the power (Caldas *et al.* 2022; Elise Lauterbur *et al.* 2022). For SFS-based methods, a larger window size may increase the power for detecting recent selection if there is less recombination because the signature of selection remains on the broader region. On the contrary, a larger window size may cause a reduction in power for old selection because recombination events narrow down homozygous regions for selected allele. Because the relationship between the window size and power is complicated by the effects of many parameters, comprehensive investigation will be required. The detection of other types of selection, such as balancing selection (Charlesworth 2006; Fijarczyk and Babik 2015; de Filippo *et al.* 2016) and polygenic adaptation (Latta 1998; Le Corre and Kremer 2012; Berg and Coop 2014), is another issue. In the case of polygenic adaptations, it is expected that the shift in allele frequency becomes smaller than that of hard sweep (Barghi and Schlötterer 2020); therefore, it is much more difficult to detect. These issues should be also resolved in future studies.

## Supplementary Material

jkad161_Supplementary_Data

## Data Availability

Code used to generate the simulated data can be found at https://github.com/ttomo3535/power_of_neutrality_tests. Supplementary Material is available at figshare: https://doi.org/10.25387/g3.23659659.
