# Supplementary material for: Power of neutrality tests for detecting natural selection
Source: G3 (Bethesda). 2023 Jul 22;13(10):jkad161. doi: 10.1093/g3journal/jkad161 (PMC10542275; doi:10.1093/g3journal/jkad161)
Supplement: jkad161_Supplementary_Data [file jkad161_supplementary_data.docx]

**Supplement Figures**

Supplement Figure 1 The demographic model of the (A)W. African population, (B) W. European population, and (C) E. Asian population estimated by Schaffner *et al*. (2005). *N* is population size. Arrows represent the timing of demographic events in a generation. The duration of the bottleneck is 100 generations.

Supplementary Figure 2 Simplified demographic model of European and Asian populations estimated by Li and Durbin (2011). *N* is population size. Arrows represent the timing of the demographic events in years.

Supplementary Figure 3 The power of four tests under the constant population model against the allele frequency in the current population. The selection coefficient was set to be 0.001, 0.01, and 0.1 (top to bottom).

Supplementary Figure 4 The power (left columns) and false positive rate (right columns) of four tests for a range of current derived allele frequencies under a constant population model and three expansion models with *t_1_* =0.1, 0.3, and 0.5, respectively, where *t_1_* is time when expansion occurred in N generation. The selection coefficient *s* is set to be 0.005.

Supplementary Figure 5 (A) Distribution of Tajima’s *D* statistics and (B) distribution of Fay and Wu’s *H* under SNM (red) and expansion model (blue). The X-axis is the time when expansion occurred. The solid lines are 50 percentile and the shaded ranges are interquartile ranges. (C) False positive rate of two tests for a range of expansion timings. The X-axis is the time when expansion occurred.

Supplementary Figure 6 Current mean derived allele frequency against age of the derived allele under a constant population model and three expansion models with *t_1_* =0.1, 0.3, and 0.5, respectively, where *t_1_* is time when expansion occurred in N generation. (A) The selection coefficient *s* is set to be 0.005. (B) The selection coefficient *s* is set to be 0.

Supplementary Figure 7 The power (left columns) and false positive rate (right columns) of four tests against the current derived allele frequencies under a constant population model and three bottleneck models with *t_b_* =0.05, 0.2, and 0.5, respectively, where *t_b_* is the time when bottleneck ended in a unit of *N* generations. The selection coefficient *s* is set to be 0.005.

Supplementary Figure 8 The false positive rate of four tests for a range of the age of mutation under a constant population model and three bottleneck models with *t_b_* =0.05, 0.2, and 0.5, respectively, where *t_b_* is the time when bottleneck ended in *N*・generation. The selection coefficient *s* is set to be 0.005.

Supplementary Figure 9 (A) Distribution of Tajima’s *D* statistics and (B) distribution of Fay and Wu’s *H* under SNM (red) and bottleneck model (blue). The X-axis is the time when the bottleneck ended in a unit of *N* generations. The solid lines are 50 percentile and the shaded ranges are interquartile ranges. (C) False positive rate of two tests for a range of bottleneck timings. The X-axis is the time when the bottleneck ended in *N* generations.

Supplementary Figure 10 Power of four tests for a range of derived allele frequencies under models plausible for W. African, W. European, and E. Asian populations (Left to right) and under the constant population model with N = 16000, 13000, and 12000 (Left to right). The selection coefficient *s* was set to 0.005.

Supplementary Figure 11 Power and false-positive rate of four tests for a range of current-derived allele frequencies. *p* = 1/2*N* and is 0.01, 0.1, 0.2, and 0.5, respectively (left to right); it is the frequency of the derived allele when the selection started. The selection coefficient *s* was set to 0.005.

Supplement Figure 12 Power of rEHH for a range of derived allele ages under a constant population model. The dash-dot lines represent the peak age distribution of candidate genes estimated in a previous study (8000 years for the African population and 5250 years for the European population) (Hawks *et al.* 2007). The selection coefficient was set to 0.022 and 0.034, which is the average fitness advantage estimated for the European and African populations, respectively.
